# Predicting non-elective hospital readmission or death using a composite assessment of cognitive and physical frailty in elderly inpatients with cardiovascular disease

**DOI:** 10.1186/s12877-020-01606-8

**Published:** 2020-06-22

**Authors:** Si-Min Yao, Pei-Pei Zheng, Yao-Dan Liang, Yu-Hao Wan, Ning Sun, Yao Luo, Jie-Fu Yang, Hua Wang

**Affiliations:** 1 Peking University Fifth School of Clinical Medicine, P. R. China. No. 1, DaHua Road, Dong Dan, Beijing, 100730 People’s Republic of China; 2 Department of Cardiology, Beijing Hospital, National Center of Gerontology, Institute of Geriatric Medicine, Chinese Academy of Medical Sciences, P. R. China. No. 1, DaHua Road, Dong Dan, Beijing, 100730 People’s Republic of China

**Keywords:** Cognitive impairment, Frailty, Cardiovascular diseases, Non-elective hospital readmission, Elderly inpatients

## Abstract

**Background:**

We aimed to assess the utility of the combination of the mini-mental state examination (MMSE) + clock drawing test (CDT) and the Fried phenotype for predicting non-elective hospital readmission or death within 6 months in elderly inpatients with cardiovascular disease (CVD).

**Methods:**

A single-center prospective cohort was conducted from September 2018 to February 2019. Inpatients ≥65 years old were recruited. Predictive validity was tested using a Cox proportional hazards regression model analysis, and the discriminative ability was evaluated by the receiver operating characteristic (ROC) curve.

**Results:**

A total of 542 patients were included. Overall, 12% (64/542) screened positive for cognitive impairment, 16% (86/542) were physically frail and 8% (44/542) had cognitive impairment combined with physical frailty, showing an older age (*P* < 0.001) and a lower education level (*P* < 0.001) than physically frail patients. A total of 113 patients (20.9%) died or were readmitted at 6 months. Frail participants with a normal (hazard ratio [HR]: 1.73, 95% confidence interval [CI]: 1.06–2.82, *P* = 0.028) or impaired cognition (HR: 2.50, 95% CI: 1.27–4.91, *P* = 0.008) had a higher risk of non-elective hospital readmission or death than robust patients after adjusting for the age, sex, education level, marital status, the presence of diabetes mellitus, heart failure, and history of stroke. The area under the ROC curve (AUC) showed that the discriminative ability in relation to 6 months readmission and death for the MMSE + CDT + Fried phenotype was 0.65 (95% CI: 0.60–0.71), and the AUC for men was 0.71 (95% CI: 0.63–0.78), while that for women was 0.60 (95% CI: 0.51–0.69).

**Conclusions:**

Accounting for cognitive impairment in the frailty phenotype may allow for the better prediction of non-elective hospital readmission or death in elderly inpatients with CVD in the short term.

**Trial registration:**

ChiCTR1800017204; date of registration: 07/18/2018.

## Background

Cardiovascular disease (CVD) is the leading cause of death and disability [1]. Ischemic heart disease, heart failure (HF), and atrial fibrillation (AF) are cardiovascular conditions with markedly high rates of morbidity and mortality. Cardiovascular mortality in individuals 50 to 69 years of age was reported to be 436 deaths for every 100,000 people [2], increasing with age. China has the largest ageing population and is one of the fastest aging countries in the world [3]. CVD and related complications are significant healthcare problems in the growing elderly population.

Metabolic factors are the predominant risk factors for CVD, and behavioral risk factors, a low education, and a low grip strength have strong effects on CVD or mortality [4]. Sedentary behavior and physical inactivity are major modifiable risk factors for CVD [5]. A significant association between frailty and a poor prognosis has been described in patients with CVD [6, 7]. Age-associated cognitive decline and impairment have also been shown to be associated with an increased mortality [8, 9]. However, most previous reports only evaluated the relationship between physical frailty or cognitive impairment and CVD.

In contrast, high rates of cognitive impairment have been reported among older adults with increased levels of frailty, and physical frailty and cognitive impairment often co-occur [10]. The definitions of cognitive and physical frailty and their predictive ability for an adverse outcome in elderly inpatients with CVD has not been thoroughly investigated.

Physical frailty represents a state of increased vulnerability to stressor events, weakness, risk of morbidity, disability, and mortality [11]. Cognitive frailty is a heterogeneous clinical manifestation characterized by the simultaneous presence of both physical frailty and cognitive impairment in the absence of dementia and other neurodegenerative diseases [10].

Cognitive and physical components of frailty have pathophysiologic rationale as risk factors for CVD. There is a clinical need to identify more practical screening systems that can aid in detecting patients with cognitive impairment and physical frailty, and determining which patients with CVD are at a high risk of adverse outcomes, as the early management of these high-risk patients can reduce readmission rates and healthcare spending and improve the quality of care [12].

Accordingly, the primary aim of the present study was to assess the utility of the combination of the mini-mental state examination (MMSE) + clock drawing test (CDT) and the Fried phenotype for predicting non-elective hospital readmission or death within 6 months in elderly inpatients with CVD and to assess the different predictive performance for relevant adverse outcome of the following domains: cognitive function, physical performance, and nutritional status. We also performed a sensitivity analysis substituting the Short Physical Performance Battery (SPPB), another more objective measure of simple physical condition, for the Fried phenotype.

## Methods

### Participants

Inpatients ≥65 years old who were admitted to the Department of Cardiology, from September 2018 to February 2019 were recruited. Among these patients, 746 eligible patients were enrolled, and inappropriate patients were excluded for the following reasons: unable to cooperate with the questionnaires and follow-up for various reasons (*n* = 17), did not agree to undergo the assessments (*n* = 175), and quit the test ahead of schedule (*n* = 12). A total of 204 patients were excluded, and 542 were enrolled into the final analyses. All patients enrolled in the study were followed after discharge. During the six-month follow-up, the researchers followed the patients mainly through outpatient visits and telephone calls. There were no patient lost to follow-up (Supplementary Table S1).

### Definitions of the four groups

Physical frailty was defined according to the definition proposed by Fried et al. based on the five criteria of unintentional weight loss, self-reported exhaustion, weakness, slow walking speed, and low physical activity [11]. Participants were ranked as frail (3–5 criteria), or non-frail, including prefrail (1 or 2 criteria) and robust (0 criteria).

We used the Chinese version of the MMSE and CDT to define cognitive impairment: (1) below 24 points of MMSE or (2) 24 ≤ MMSE ≤26 and incorrect CDT.

A four-level composite frailty scoring system was created via the combination of the cognition impairment and frailty [13], and the present study is the first to formally use the MMSE + CDT to assess the cognition status. Robust patients (RP) were non-frail without cognitive impairment, cognitive impairment (CI) patients were non-frail with cognitive impairment, physical frailty (PF) patients were frail without cognitive impairment, and cognitive frailty (CF) patients were frail with cognitive impairment.

### Outcome measures

The primary outcome for this study was the non-elective hospital readmission or death at 6 months, with the former considered any type of readmission, such as emergency visits, or an urgent admission requested by the general practitioner [14], and the latter referring to death for any reason.

### SPPB

The simple physical condition was measured by the SPPB, scored from 1 to 12 based on three tests: a set of balance tests, gait speed and repeated chair stands. The SPPB (cut-off value of 10) is an effective assessment tool for measuring the lower extremity function for middle-aged and older CVD patients that is widely used in both clinical and research settings [15, 16].

### Statistical analyses

In this study, descriptive statistics were calculated for all variables. Continuous variables were expressed as the mean ± standard deviation (SD) in a normal distribution and median and interquartile range in a non-normal distribution, and categorical variables were expressed as numbers and percentages. We used the chi-square test for categorical data and a one-way analysis of variance for continuous data to compare the differences between groups. The correlation between cognitive impairment and HF and between frailty and HF was analyzed by the chi-square test. The Kaplan-Meier curves with log-rank tests were used to estimate the cumulative incidence of events. The Cox proportional hazards regression model to estimate hazard ratio (HR) with 95% CI was used to analyze the association between the frailty status or other factors at baseline and the non-elective hospital readmission or death. The Cox regression was adjusted for the age, sex, education level, marital status, the presence of HF, diabetes mellitus (DM) and history of stroke. The areas under the receiver operating characteristics (ROC) curve were used to evaluate the discriminative ability of the MMSE, MMSE + CDT, Fried phenotype, MMSE + CDT + Fried phenotype and MMSE + CDT + Fried phenotype + mini nutritional assessment-short form (MNA-SF) in relation to composite endpoints including readmission and death. The area under the ROC curve (AUC) was determined to measure the accuracy of the prediction.

A *P*-value < 0.05 was considered statistically significant. All the data analyses were conducted using the IBM SPSS Statistics software program (version 24; IBM Corporation, Armonk, NY, USA).

### Covariates

Several potential confounders and effect modifiers were measured and defined. Sociodemographic characteristics were age, sex, marital status (married or non-married, including single, divorced, separated and widowed), education level, and body mass index (BMI). Lifestyle behaviors were smoking status (yes or no, including quitting smoking in the last 3 months), alcohol intake (yes or no, including quitting drinking in the last 3 months). Health status was the medical history, including hypertension, coronary atherosclerotic heart disease (CAD), AF, HF, DM, and history of stroke. Laboratory indicators included the serum free triiodothyronine (FT3) and prealbumin (PA).

Regarding the nutritional status, the MNA-SF was validated for the diagnosis of malnutrition and prediction of clinical outcomes, including six items with a total score of 14. Patients were divided into three categories: 12–14 points indicated “well-nourished”, 8–11 points indicated “at risk of malnutrition”, and 0–7 points indicated “malnourished” [17]. All covariate information was obtained using a standardized and structured questionnaire in the baseline survey, and venous blood samples were collected in the early morning from fasting patients.

## Results

### Table 1. Characteristics of clinical data in the general population

Overall, the mean (SD) age was 75.17 (6.52) years old at baseline, and 48.5% of participants (263/542) were women with a mean BMI of 25.25 (3.37) kg/m^2^. Of the total participants, 64% (348/542) were classified as RP, 12% (64/542) as CI, 16% (86/542) as PF, and 8% (44/542) as CF. The most frequent hospital admission diseases were hypertension (73.1%), CAD (59%), DM (34.8%), AF (22.2%), history of stroke (16.7%), and HF (12.2%).

**Table 1:** The baseline characteristics of the four-level composite frailty scoring system

	RP (*n* = 348)	CI (*n* = 64)	PF (*n* = 86)	CF (*n* = 44)	*P value*
Age (year)	73.33 ± 5.73	78.87 ± 6.21*	76.62 ± 6.73*	81.65 ± 5.34*^‡^	< 0.001^a^
Women	156 (44.8%)	35 (54.7%)	43 (50.0%)	29 (65.9%)	< 0.05^b^
Non-married	44 (12.6%)	21 (32.8%)*	16 (18.6%)	15 (34.1%)*	< 0.001^b^
Education (year)	11.89 ± 3.65	8.55 ± 4.9*	12.06 ± 3.54^†^	7.07 ± 4.78*^‡^	< 0.001^a^
BMI (kg/m^2^)	25.28 ± 3.31	25.41 ± 3.61	25.58 ± 3.43	24.07 ± 3.23	0.095^a^
Smoke	123 (35.3%)	17 (26.6%)	20 (23.3%)	13 (29.5%)	0.125^b^
Alcohol intake	130 (37.4%)	16 (25.0%)	20 (23.3%)	8 (18.2%)	< 0.05^b^
Hypertension	248 (71.5%)	50 (79.4%)	61 (70.9%)	36 (81.8%)	0.303^b^
CAD	197 (56.6%)	36 (56.0%)	48 (55.8%)	21 (44.7%)	0.749^b^
AF	61 (17.6%)	20 (31.7%)	23 (26.7%)	16 (36.5%)	< 0.05^b^
HF	26 (7.5%)	13 (20.6%)	17 (19.8%)	10 (22.7%)	< 0.001^b^
DM	106 (30.5%)	25 (39.7%)	40 (46.5%)	17 (38.6%)	< 0.05^b^
History of stroke	45 (13.0%)	13 (20.6%)	17 (19.8%)	15 (34.1%)*	< 0.05^b^
SPPB< 10	188 (54.0%)	55 (85.9%)*	78 (90.7%)*	41 (93.2%)*	< 0.001^b^
MNA-SF(< 12)	86 (24.7%)	20 (31.2%)	44 (51.2%)*^†^	23 (52.3%)*	< 0.001^b^
MMSE	28.32 ± 1.56	22.14 ± 4.43*	28.06 ± 1.57^†^	19.48 ± 5.64*^†‡^	< 0.001^a^
CDT (Incorrect)	77 (22.1%)	52 (82.5%)*	22 (25.6%)^†^	36 (81.8%)*^‡^	< 0.001^b^
PA (mg/dl)	25.31 ± 5.84	23.64 ± 5.10	22.71 ± 5.08*	19.82 ± 5.60*^†^	< 0.001^a^
FT3 (pg/ml)	3.17 ± 0.39	3.06 ± 0.45	2.96 ± 0.36*	2.87 ± 0.36*	< 0.001^a^

Values are showed as mean ± standard deviation or *n* (%)

^a^One-way analysis of variance

^b^Chi square test

*RP* robust patients, *CI* cognitive impairment, *PF* physical frailty, *CF* cognitive frailty, *BMI* body mass index, *CAD* coronary atherosclerotic heart disease, *AF* atrial fibrillation, *HF* heart failure, *DM* diabetes mellitus, *SPPB* short physical performance battery, *MNA-SF* mini nutritional assessment-short form, *MMSE* mini-mental state examination, *CDT* clock drawing test, *PA* prealbumin, *FT3* free triiodothyronine

*P < 0.001 compared with RP

^†^*P* < 0.001 compared with CI

^‡^*P* < 0.001 compared with PF

### A comparison of the clinical data among the four groups

There were no marked differences in the BMI, smoke, hypertention, and CVD (*P* > 0.05) among the four groups. Those who were older (*P* < 0.001) with a lower education level (*P* < 0.001) tended to exhibit CF rather than PF, and the PA level (*P* = 0.002) was lower than that of CI. After grouping marital status by gender, the proportion of non-married women was higher than that of non-married men (27.4%; 8.6%; *P* < 0.05), especially in the CI (40.0%; 24.1%; *P* < 0.05) and CF (51.6%; 0.0%; *P* < 0.05) groups.

### Interaction between cognitive and HF and between frailty and HF

The prevalence of physical frailty (25.8%: 17/66; 14.5%: 69/476; *P* = 0.019) and cognitive frailty (15.2%: 10/66; 7.1%: 34/476; *P* = 0.026) were much more common in HF patients than in Non-HF subjects. The prevalence of cognitive impairment (19.7%: 13/66; 10.7%: 51/476; *P* = 0.034) was higher in patients with HF than Non-HF. There were marked differences in the MMSE and Fried phenotype (*P* < 0.001) among the two groups (HF vs. Non-HF). The mean MMSE score (SD) was 24.38 (6.07) in patients with HF, compared with 27.17 (3.39) in Non-HF patients. The mean Fried phenotype (SD) was 1.51 (1.20) in patients with HF, compared with 2.39 (1.14) in Non-HF patients. HF is slightly related to physical frailty (kappa = 0.10), cognitive impairment (kappa = 0.09) and cognitive frailty (kappa = 0.09) using the Chi-square test (Supplementary Table S2).

### Follow-up events

**Fig. 1:**
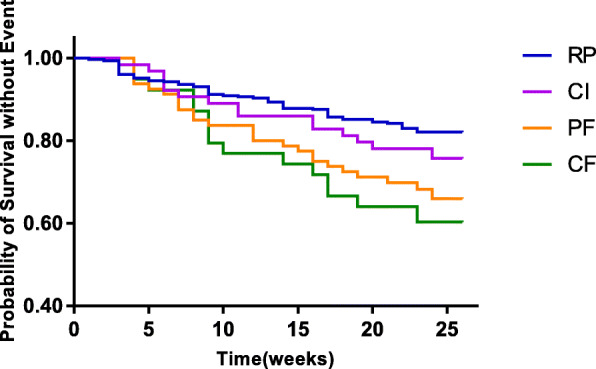
Kaplan-Meier survival curves by cognitive impairment and frailty status (*n* = 542). Patients were divided into groups of RP, CI, PF and CF via the combination of the MMSE + CDT and Fried phenotype, which were significantly different (log-rank χ^2^ = 15.78; *P* < 0.001). Pairwise comparisons with adjustment for multiple comparisons demonstrated significant differences between the CF and RP groups (χ^2^ = 10.54;*P* < 0.001), and PF and RP groups (χ^2^ = 9.24;*P* = 0.002), although there was no significant difference between the CI and RP groups (χ^2^ = 1.21;*P* = 0.27). RP, robust patients; CI, cognitive impairment; PF, physical frailty; CF, cognitive frailty; MMSE, mini-mental state examination; CDT, clock drawing test

During the follow-up, there were 113 events (4 deaths and 109 non-elective hospital readmissions). The clinical outcome of non-elective hospital readmission and death occurred with increased frequency in the four groups, as follows: 16% (57/348), 23% (15/64), 30% (26/86), and 34% (15/44) of patients with respective MMSE + CDT + Fried phenotype subcategories of RP, CI, PF, and CF (χ^2^ = 13.74; *P* = 0.003). The Kaplan-Meier curve shows the same trend (Fig. 1) (log-rank χ^2^ = 15.78; *P* < 0.001). The rates of surviving without events in the PF (χ^2^ = 9.24; *P* = 0.002) and CF (χ^2^ = 10.54; *P* = 0.001) groups were higher than in the RP group, but there was no significant difference between the CI (χ^2^ = 1.21; *P* = 0.27) and RP groups.

**Fig. 2:**
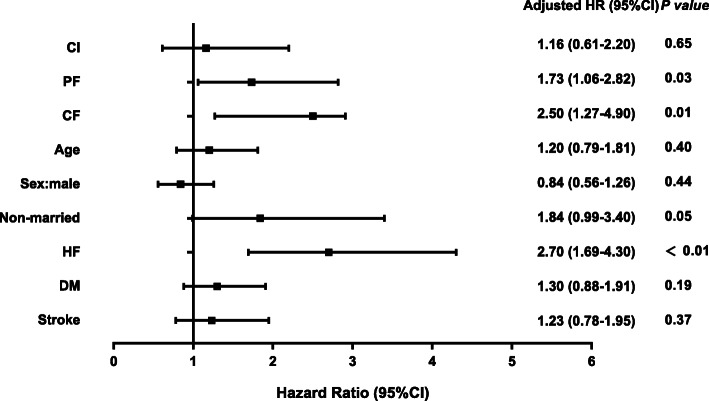
Cox proportional hazards regression model: the risk factors for the non-elective hospital readmission or death (*n* = 542). Age was a categorical variable: 65–74; 75–84; ≥85 years old. CI, cognitive impairment; PF, physical frailty; CF, cognitive frailty; HF, heart failure; DM, diabetes mellitus; HR, hazard ratio; 95% CI, 95% confidence interval

The Cox proportional hazards regression model was used to analyze the risk factors for non-elective hospital readmission or death. After adjusting for the age, sex, education level, marital status, presence of DM, HF, and history of stroke, PF and CF were independently significant predictors of non-elective hospital readmission or death (HR: 1.73, 95% CI: 1.06–2.82, *P* = 0.028; HR: 2.50, 95% CI: 1.27–4.91, *P* = 0.008) in elderly inpatients with CVD (Fig. 2).

### Sensitivity analysis

**Fig. 3:**
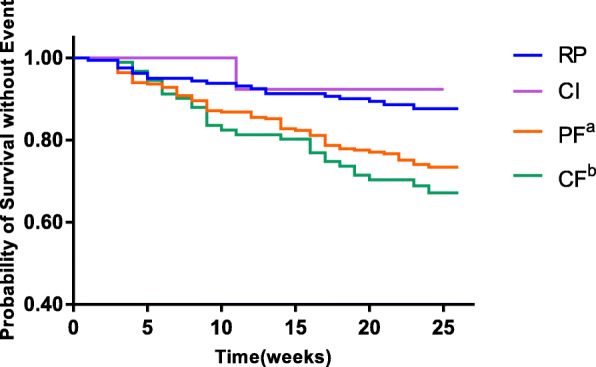
Kaplan-Meier survival curves by cognitive impairment and SPPB status (*n* = 542). Patients were divided into groups of RP, CI, PF^a^ and CF^b^ via the combination of the MMSE + CDT and SPPB (log-rank χ^2^ = 15.43; *P* < 0.001). Pairwise comparisons with adjustment for multiple comparisons demonstrated significant differences between the CF^b^ and RP groups (χ^2^ = 13.74; *P* < 0.001), and the PF^a^ and RP groups (χ^2^ = 10.93;*P* = 0.001), with no significant difference noted between the CI and RP groups (χ^2^ = 0.09; *P* = 0.760). ^a^ Lower SPPB. ^b^ Lower SPPB + cognitive impairment. RP, robust patients; CI, cognitive impairment; PF, physical frailty; CF, cognitive frailty; SPPB, short physical performance battery; MMSE, mini-mental state examination; CDT, clock drawing test

A sensitivity analysis was performed for the association between the MMSE + CDT + SPPB and non-elective hospital readmission or death. The Kaplan-Meier curve also showed that the rates of surviving without events in the PF (lower SPPB) (χ^2^ = 10.93; *P* = 0.001) and CF (lower SPPB + cognition impairment) (χ^2^ = 13.74; *P* < 0.001) groups were higher than in the RP group, but there was no significant difference between the CI (χ^2^ = 0.09; *P* = 0.760) and RP groups (Fig. 3). In the fully adjusted Cox proportional hazards regression model, lower SPPB and lower SPPB + cognition impairment were independently associated with non-elective hospital readmission or death respectively (HR: 2.25, 95% CI: 1.32–3.81, *P* = 0.001; HR: 2.64, 95% CI: 1.34–5.20, respectively; *P* < 0.001). Both the Fried phenotype assessment and SPPB test were performed safely, even in patients with various chronic diseases. In the present study, there were no adverse events caused by the assessments or tests.

### Predictive capacity

ROC analyses (Table 2) were used to evaluate the ability of MMSE, MMSE + CDT, Fried phenotype, MMSE + CDT + Fried phenotype and MMSE + CDT + Fried phenotype + MNA-SF to discriminate cases of six-month readmission and death among all individuals. The AUC showed a discriminative ability relative to six-month readmission and death for the MMSE + CDT + Fried phenotype of 0.65 (95% CI: 0.60–0.71), and this value in men was 0.71 (95% CI: 0.63–0.78), at which the sensitivity was 70.3% and the specificity was 61.9%, while the value in women was 0.60 (95% CI: 0.51–0.69). In all patients, the ability to discriminate readmission and death by MMSE alone was insufficient and showed a negative ability (AUC = 0.41), but after combining with CDT, the discriminative ability was corrected and improved (AUC = 0.59, 95% CI: 0.53–0.65). Adding MNA-SF to MMSE + CDT + Fried phenotype did not increase the predictive value.

**Table 2:** The area under the ROC curve with the 95% CI for MMSE, MMSE + CDT, Fried phenotype, MMSE + CDT + Fried phenotype and MMSE + CDT + Fried phenotype + MNA-SF

Total			Men			Women		
Index	AUC	95% CI	Index	AUC	95% CI	Index	AUC	95% CI
MMSE	0.41	0.35–0.47	MMSE	0.36	0.29–0.44	MMSE	0.46	0.37–0.55
MMSE + CDT	0.59	0.53–0.65	MMSE + CDT	0.64	0.56–0.72	MMSE + CDT	0.50	0.41–0.59
Fried phenotype	0.64	0.58–0.70	Fried phenotype	0.68	0.61–0.75	Fried phenotype	0.60	0.51–0.69
MMSE + CDT + Fried phenotype	0.65	0.60–0.71	MMSE + CDT + Fried phenotype	0.71	0.63–0.78	MMSE + CDT + Fried phenotype	0.60	0.51–0.69
MMSE + CDT + Fried phenotype + MNA-SF	0.65	0.60–0.71	MMSE + CDT + Fried phenotype + MNA-SF	0.71	0.63–0.78	MMSE + CDT + Fried phenotype + MNA-SF	0.60	0.51–0.69

*ROC* receiver operating characteristic, *CI* confidence interval, *MMSE* mini-mental state examination, *CDT* clock drawing test, *MNA-SF* mini nutritional assessment-short form

The differences in the predictive performance of a single or combination of domains showed that the predictive value in men was higher than that in women. In women, MMSE + CDT had no predictive value (AUC = 0.50).

## Discussion

Our study is one of the first to evaluate the impact of the physical and cognitive status on the risk of subsequent events in elderly patients hospitalized for CVD. Overall, 12% of elderly inpatients with CVD were diagnosed with CI, 16% with PF, and 8% with CF. Those who were older with a lower education level tended to exhibit CF rather than PF, and the PA level was lower than that of CI. We found that elderly patients with HF, DM, a history of stroke, non-married status, severe MNA-SF, and low FT3 levels at baseline were more likely to have adverse events than others, but after adjusting for confounders, only HF, cognitive frailty, severe MNA-SF and physical frailty were useful predictors of the short-term prognosis. The significance of physical frailty assessed by the Fried phenotype for predicting non-elective hospital readmission or death within 6 months in elderly patients with CVD increased after the diagnosis of cognitive impairment was added. A sensitivity analysis for the association between the MMSE + CDT + SPPB confirmed these results.

The detection of frailty in older patients with CVD is essential for the clinical management and marking therapeutic decisions. General frailty is a multidimensional construction, including physical, mental, social, nutritional and other domains. The most commonly used frailty assessment tools are the Frailty Index, the Clinical Frailty Scale (CFS), and the Fried phenotype [18]. Several studies have shown that the Frailty Index has a higher predictive ability of adverse clinical outcomes than other frailty measurements in both hospital and community settings, and it has a good validity including all concepts of frailty; however, its greatest limitation is its time-consuming nature [19]. The CFS is a rather simple semi-quantitative assessment tool based on clinical judgement but lacks an objective measurement of mobility, muscle strength, and any indices of nutritional status, which might reduce its reproducibility and usability [20]. Therefore, the Fried phenotype, which is a brief interview combined with simple physical tests that is easy to administer, seems to be the most acceptable choice; it is also used for the Cardiovascular Health Study scale [11], which is more suitable for assessing CVD patients. Unfortunately, the Fried phenotype does not include psychosocial components of frailty [19]. In the assessment of generally frail patients, adding appropriate cognitive assessment tools to the Fried phenotype may be important and useful.

Mild cognitive impairment can be described as a transition phase between normal aging and dementia. CVD affects the development of cognitive impairment in the elderly. Thrombo-embolic and/or reduction of cardiac output appear to be the main mechanisms involved in the determination of cognitive impairment in elderly patients with CVD, which involves common diseases such as CAD, hypertension, DM and HF [21, 22]. Cognitive impairment is a risk factor for adverse events in patients with CVD [8]. The most widely used tool for evaluating cognitive impairment is the MMSE [23]. However, the MMSE is language-based and considered to be influenced by the level of education. The CDT is one of the most widely used cognitive screening instruments for dementia [24], and it can be performed without being influenced by the patient’s level of language or education and is less affected by depression than other tests [25]. To our knowledge, the present study is the first to formally assess the MMSE + CDT as a predictor of clinically relevant outcomes in patients with CVD.

We found that cognitive impairment alone cannot predict non-elective hospital readmission or death in elderly inpatients with CVD, showing this ability only when combined with physical frailty. There are several possible reasons for the effect of cognitive frailty on adverse events. First, patients in the CF group had a high proportion of multiple diseases coexisting and more serious basic diseases than those in the CI group. Second, cognitive frailty patients have great difficulty with self-care management and compliance [21]. Third, the lack of social support was apparent among the cognitive frailty subjects, and social support has a proximal relationship with depressive mood, anxiety, impatience, and behavioral suppression, which may reduce an individual’s desire to participate in social activities and impede their access to a necessary social support system, thereby resulting in an increased risk of disabilities among older adults [26]. Fourth, sarcopenia is closely related to cognitive frailty. Sarcopenia is a progressive and generalized skeletal muscle disorder involving the accelerated loss of muscle mass and function that is associated with increased adverse outcomes [27]. Sarcopenia and physical frailty are closely related and contribute to cognitive frailty. In fact, many of the adverse outcomes of physical frailty may be mediated by sarcopenia, which may be considered the biological substrate for the development of frailty [28]. Cognitive impairment reinforces the neuronal changes in the central nervous system leading to changes in the levels and activity of neurotransmitters, which lead to a reduction in motor units and in the ability to maintain muscle activation, which might be related to sarcopenia [29].

Patients with HF had a greater proportion of frality and cognitive impairment, and there was a slightly correlation between frailty and HF and between cognitive impairment and HF. Cacciatore et al. also found HF is associated with cognitive impairment in older subjects [22]. HF was useful predictor of the short-term prognosis in our study. Recently, an analysis of two HF trials showed that frailty is highly prevalent in HF with reduced ejection fraction and associated with higher risk of hospitalization and death [30]. HF is the terminal manifestation of various heart diseases and is strongly affects physical performance [31]. As frailty reflects a number of damages to multiple systems, a multi-faceted and multi-system treatment may be reasonable. At present, there is no effective drug treatment for frailty. However, a recent study showed that Angiotensin Receptor-Neprilysin Inhibitor could improve physical frailty in patients with advanced HF in waiting list for heart transplantation [32].

We described the values of AUC in MMSE, MMSE + CDT, Fried phenotype, MMSE + CDT + Fried phenotype, and MMSE + CDT + Fried phenotype + MNA-SF, in order to assess the differing effects of the domains on rehospitalization and death. Regarding the AUC values, MMSE alone was insufficient to discriminate readmission, which was inconsistent with previous findings [33, 34]. The AUC value obtained for MMSE + CDT in all patients was 0.59. The CDT and MMSE reflect different cognitive characteristics, which may have affected the results [25]. The MMSE and CDT are both relatively fast, simple, and useful tools for screening cognitive impairment. The diagnostic accuracy may be higher when the MMSE is conducted along with the CDT than when conducted as a single test. Recent studies concluded that an indicator of frailty in routine care is related to readmission or mortality in patients [35, 36]. Vidan et al. evaluated 450 patients ≥70 years old and found frailty to be an independent predictor of 12-month readmission [37]. The AUC value obtained for the Fried phenotype in all patients was 0.64, which was consistent with previous studies [38, 39]. In a longitudinal cohort study that examined the effect of frailty phenotype and cognitive impairment on mortality in community for a 5 years, frailty and cognitive impairment (MMSE < 21) were significant predictors of mortality [40]. Accounting for cognitive impairment in the physical frailty phenotype will allow for the better prediction of non-elective hospital readmission or death in elderly inpatients with CVD in the short term.

Malnutrition and nutritional imbalance are thought to be strongly associated with the development of frailty and cognitive impairment due to both the biological and behavioral effects of diet [41]. The two main pathways to malnutrition in elderly patients are anorexia of aging and disease-related energy needs after a stressful event. The MNA-SF has been validated for the diagnosis of malnutrition and prediction of clinical outcomes. In our study, frailty with a normal or impaired cognition was associated with a poor nutritional status. We also found that a malnourished status which was assessed by severe MNA-SF (≤7), was associated with an increased risk of non-elective hospital readmission or death in elderly inpatients with CVD. However, adding MNA-SF to MMSE + CDT + Fried phenotype did not increase the predictive value. The physical and cognitive component domains are the main aspects to consider when evaluating generally frail patients, while the nutritional domain assessment may play a secondary role in predicting non-elective hospital readmission or death in the short term.

In the present study, the suitability of combining MMSE and CDT for identifying individuals with adverse outcomes was examined. Our results showed that MMSE + CDT was not ideal for discrimination among total patients. However, it was relatively useful when combined additionally with the Fried phenotype for identifying adverse outcomes in older male patients with CVD. The AUC value obtained for the MMSE + CDT + Fried phenotype in all patients was 0.65, and the AUC for men was 0.71, while that for women was 0.60.

We believe that several factors contributed to these gender differences. First, the number of elderly non-married women was greater than that of men, especially in the CI and CF groups; the loss of a spouse is associated with a marked decline in memory in older adults and has an independent effect on memory functioning [42], which causes them to further lose the support of their families and makes it less convenient for them to be hospitalized and seek medical advice. Second, elderly women are less economically independent, which leads to difficulty in seeking medical help, and their exposure to stimulating environments might be limited. Finally, some studies have shown that women with cognitive impairment have greater longitudinal rates of cognitive and functional progression than men, the conjecture being that this might induce changes in carers’ attitudes to protect the women [43]. Further research on gender differences and adverse outcomes should be performed in the future.

### Study limitations

Several limitations associated with the present study warrant mention. First, this was a cross-sectional study with a short-term follow-up, and there were four deaths, so the guidance concerning the short-term prognosis focused on the non-elective hospital readmission; however, a longer-term follow-up study in this population is currently being conducted by our group. Second, these data were collected from patients at one hospital, so the results may not necessarily be directly transferable to patients from different locations, although this approach allowed for the more convenient follow-up of these patients. Third, the study only included hospitalized patients, so nothing can be inferred from this study about elderly people living in the community. In addition, the small sample size is the main reason why we did not conduct a subgroup analysis of single diseases. Fourth, with the limited length of stay, we did not have an accurate assessment of sarcopenia. Finally, the combination of MMSE and CDT is not a very sensitive means of detecting subtle impairments of cognitive function, so we may have underestimated the proportion of people with cognitive impairment in the present study.

## Conclusions

Physical and cognitive frailty were powerful predictors of non-elective hospital readmission or death within 6 months in elderly inpatients with CVD, and accounting for cognitive impairment in the frailty phenotype may allow for the better prediction of adverse outcomes due to frailty in the short term. Understanding the role of cognitive impairment and frailty in the process of aging will help identify elderly inpatients at risk of adverse outcomes and provide reasonable nursing care and management. Learning how the physical and cognitive status affects the prognosis, and determining the specific processes and possible mechanisms involved, we can systematically identify and develop care pathways for older people with cognitive frailty and take new and appropriate interventions to reduce their health risk.

## Supplementary information

**Additional file 1: Supplementary Table S1.** Flow diagram of patient selection.

**Additional file 2: Supplementary Table S2.** Interaction between cognitive impairment and HF and between frailty and HF.

## Data Availability

The datasets used and/or analyzed during the current study are available from the corresponding author on reasonable request.

## Abbreviations

CVD: Cardiovascular disease
HF: Heart failure
AF: Atrial fibrillation
MMSE: Mini-mental state examination
CDT: Clock drawing test
SPPB: Short physical performance battery
RP: Robust patients
CI: Cognitive impairment
PF: Physical frailty
CF: Cognitive frailty
SD: Standard deviation
HR: Hazard ratio
95% CI: 95% confidence interval
DM: Diabetes mellitus
ROC: Receiver operating characteristic
AUC: Areas under the curve
BMI: Body mass index
CAD: Coronary atherosclerotic heart disease
FT3: Free triiodothyronine
PA: Prealbumin
MNA-SF: Mini nutritional assessment-short form
CFS: Clinical Frailty Scale
