# Supplementary figures and images for: Predicting non-elective hospital readmission or death using a composite assessment of cognitive and physical frailty in elderly inpatients with cardiovascular disease
Source: BMC Geriatr. 2020 Jun 22;20:218. doi: 10.1186/s12877-020-01606-8 (PMC7309999; doi:10.1186/s12877-020-01606-8)

**Supplementary Table S1:** Flow diagram of patient selection.


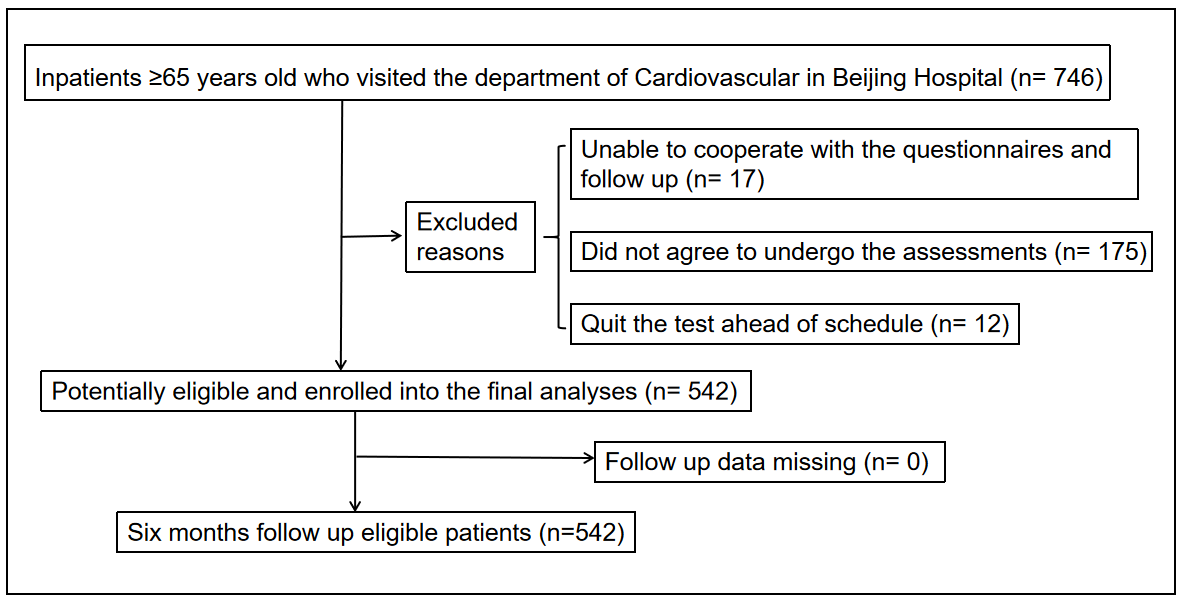

Supplement: Supplementary file 1 — Additional file 1: Supplementary Table S1. Flow diagram of patient selection. [file 12877_2020_1606_MOESM1_ESM.doc]
