# Supplementary material for: Predicting non-elective hospital readmission or death using a composite assessment of cognitive and physical frailty in elderly inpatients with cardiovascular disease
Source: BMC Geriatr. 2020 Jun 22;20:218. doi: 10.1186/s12877-020-01606-8 (PMC7309999; doi:10.1186/s12877-020-01606-8)
Supplement: Supplementary file 2 — Additional file 2: Supplementary Table S2. Interaction between cognitive impairment and HF and between frailty and HF. [file 12877_2020_1606_MOESM2_ESM.doc]

***Supplementary Table S2:*** *Interaction between cognitive impairment and HF and between frailty and HF.*

|  | **HF**  **(n=66)** | **Non-HF**  **(n=476)** | ***P***  ***value*** |
| --- | --- | --- | --- |
| Cognitive impairment | 13 (19.7%) | 51 (10.7%) | 0.034^b^ |
| Physical frailty | 17 (25.8%) | 69 (14.5%) | 0.019^b^ |
| Cognitive frailty | 10 (15.2%) | 34 (7.1%) | 0.026^b^ |
| MMSE | 24.38 (6.07) | 27.17 (3.39) | <0.001^a^ |
| CDT (Incorrect) | 33 (50.0%) | 154 (32.5%) | 0.005^b^ |
| Fried phenotype | 2.39 (1.14) | 1.51 (1.20) | <0.001^a^ |

Values are showed as mean ± standard deviation or n (%). ^a^One-way analysis of variance; ^b^Chi square test. MMSE, mini-mental state examination; CDT, clock drawing test.
